# The impact of the pilot reform of home and community-based elderly care services on the health of the elderly

**DOI:** 10.1186/s12889-026-26244-4

**Published:** 2026-01-19

**Authors:** Han Qiu, Xiaoxuan Yang, Yuying Zhu, Yi Zhang

**Affiliations:** 1 Dong Fureng institute of economics and social development, Wuhan university, Wuhan, Hubei 430072 China; 2 School of Labor Economics of Capital University of Economics and Business, Beijing, 100070 China

**Keywords:** Home and community-based elderly care services, Mental and functional health, Older adults, Accessibility of elderly care services, Evidence-based policy

## Abstract

**Background:**

The Home and Community-based Elderly Care Services (HCBS) pilot aims to improve access to preventive, rehabilitative, and long-term care for a rapidly ageing population. This study estimates the effect of HCBS on elderly mental and functional health and examines mechanisms and distributional heterogeneity

**Methods:**

This paper linked the roll-out of HCBS pilots (2016–2020) to five waves (2011–2020) of the China Health and Retirement Longitudinal Study (CHARLS). The analytic sample included 25,287 respondents aged ≥60 years. This paper implemented a multi-period staggered difference-in-differences (DID) with individual and year fixed effects to estimate impacts on depressive symptoms (10-item CESD, 0–30; higher = worse) and physical status (ADL, 0–11; higher = worse). Mechanisms were tested using community elderly care intensity proxies, including home physician visits, family-doctor contracting, and routine preventive care.

**Results:**

The HCBS significantly improved both mental and functional health. CESD scores declined by 0.396 (≈4.53% of the mean) and ADL scores by 0.224 (≈20.1% of the mean). Mechanism analysis showed increased use of routine preventive care (2.52 percentage points) and higher participation in home visits and family-doctor contracting, consistent with enhanced accessibility of elderly care services. Robustness checks found no differential pre-trends; effects were driven primarily by treated vs. never-treated comparisons (Goodman-Bacon weight ≈0.96), and remained significant under plausible deviations from parallel trends. Larger CESD improvements among those without chronic conditions and with higher education, and larger ADL gains among those with chronic conditions, lower education, and rural residents.

**Conclusions:**

The HCBS significantly improved the mental and functional health of the elderly through enhancing the accessibility of community elderly care services. Prioritizing service continuity and tailoring supports to functionally vulnerable groups could further amplify health gains and reduce disparities.

**Clinical trial number:**

Not applicable.

**Supplementary Information:**

The online version contains supplementary material available at 10.1186/s12889-026-26244-4.

## Introduction

China has entered the stage of rapid population aging, and it’s an unavoidable issue about addressing how to efficiently support the elderly [1].” Raise children to provide against old age” is an essential feature of the Chinese traditional approach to eldercare. Under the influence of Confucianism, the family-based care is deep-rooted [2]. Historically, the elderly care challenges have been predominantly addressed through intergenerational financial transfers from offspring over extended periods [3]. However, various factors such as " age before it gets rich”, “Fertility decline”, and “Functional erosion of familial elderly support " are challenging this traditional model [4]. Community elderly care and institutional elderly care also face high costs and low coverage rates. Consequently, the traditional models of home elderly care, community elderly care, and institutional elderly care are insufficient to meet the needs of the elderly. Only by adopting a coordinated approach that integrates home-based, community-based, and institutional care can we meet the demands of a rapidly aging society [5]. Against this backdrop, China advanced the pilot reform of home and community-based elderly care services(HCBS). In 2016, the Ministry of Civil Affairs and the Ministry of Finance jointly launched the pilot to enable the vast majority of older adults with care needs to receive formal elderly-care services at home through community platforms and service institutions. From 2016 to 2020, the pilot was implemented in five cohorts, with the central government allocating RMB 1 billion annually, approximately RMB 5 billion in total, across 203 pilot localities, covering all provinces, autonomous regions, and municipalities directly under the central government.

The HCBS emphasizes the coordination between primary health care and elderly care services, promotes community-based elderly care and encourages private enterprises to participate in service provision. Through lowering informational, temporal, and financial barriers, preventive, managerial and rehabilitative services can be shifted closer to the elderly, thereby transforming the traditional service structure that has long been dominated by acute treatment [6]. Theoretically, this aligns with the logic of the health production function, in which improved accessibility and reduced marginal acquisition costs of elderly care services facilitate utilization, ultimately enhancing both mental and functional health.

Scholars have conducted research on the HCBS. The HCBS have significantly improved the mental and functional health of the elderly. Additionally, the policies positively influence their life satisfaction and well-being [7–9]. This is because this policy provides medical and care services for the elderly, enhancing filial support for the elderly and promoting social participation of the elderly. Firstly, this policy provides professional medical and care services for the elderly [7], such as regular health check-ups and chronic disease management, which is conducive to elderly access continuous and accessible health services under the concept of proactive health [10], favorable to effectively reducing disease burden, lowering disability risks, and enhancing health and sense of happiness in later life [11], propitious for improve elderly health management situation, cultivate healthy lifestyles, and ultimately promote overall health outcomes [12]. Secondly, this policy could enhance filial support for the elderly, including economic assistance and spiritual comfort [8]. The support from children could improve the functional health and mental health of the elderly, significantly increasing the sense of happiness. This improvement is more pronounced among older elderly individuals [13]. Thirdly, this policy could promote social participation of the elderly [7]. The participation of older adults in social activities facilitates clearer self-perception, thereby alleviating negative emotions caused by role loss while enhancing life satisfaction and sense of happiness. This process helps the elderly to maintain vitality and engagement across physical, psychological, and social dimensions [14]. Furthermore, social engagement significantly improves the elderly overall health status by mitigating impairments in basic activities of daily living (ADL), instrumental activities of daily living (IADL), and cognitive function, while simultaneously reducing the incidence risk of chronic diseases [15, 16].

The HCBS was implemented in a staggered manner across different cities between 2016 and 2020. Accordingly, this study adopts a multi-period dynamic difference-in-differences (DID) approach to evaluate policy implementation and its effects. Specifically, the policy list of pilot regions is matched with five waves of data (2011–2020) from the China Health and Retirement Longitudinal Study (CHARLS). A multi-period DID and event-study design is constructed to estimate the causal impact of the reform on mental health (measured by the CESD scale) and functional health (measured by the ADL scale). Furthermore, community-based service utilization, including home visits by physicians, family doctor contracting and annual physical examinations, is employed as an operational mediator for “community care intensity” to test the mechanism channel of “Pilot reforms lead to improved service accessibility, which in turn results in better health outcomes”. Building on this, heterogeneity analyses are conducted along three dimensions, including disease burden (chronic condition status), educational attainment, and rural–urban residence, to explore “who benefits and why the effects differ”. It provides an evidence base for stratified and targeted policy refinement.

Compared with existing studies, there are three marginal contributions in this paper. Firstly, previous research has mainly focused on the demand side of older adults, which means enhancing their purchasing power through long-term care insurance and consumption subsidies for age-friendly home adaptation products. In contrast, this paper examines the policy effects of the HCBS, which emphasizes improving the accessibility of services, namely by expanding the supply of elderly care. The HCBS enhances the supply of universal community and home-based care by improving service infrastructure, innovating service content, and deepening the integration of medical care, rehabilitation and social care. Therefore, this study investigates the health effects of the HCBS pilot from the perspective of service supply. Secondly, existing studies often rely on a single indicator to measure the health of the elderly when assessing the impact of HCBS, which is inherently limited. Different dimensions of health are intrinsically interrelated, and a single indicator cannot capture the interactions among these dimensions. In addition, a unidimensional measure may obscure the true distribution of health problems. This paper constructs a combined measure based on the CESD and ADL indices to provide a multidimensional assessment of health, which better captures the complexity of health status and avoids the evaluation bias associated with single indicators, thereby offering a more precise basis for designing targeted support measures. Besides, this study employs a series of cutting-edge robustness measures, including Bacon Analysis, Sensitivity analysis of parallel trends, and Heterogeneity treatment effect. The results show that the core findings remain highly consistent across these different tests. Finally, on the theoretical side, this paper explicitly embeds the “accessibility of elderly care services” into Grossman’s health-capital model. It analytically decomposes the policy effect into a direct effect and three indirect channels (price, time, and information), which are largely absent from existing studies. This paper explicitly connects the accessibility mechanism to distributional heterogeneity: the model predicts that information effects dominate for healthier, better-educated elders (primarily improving mental health), whereas price/time effects dominate for chronically ill, low-educated and rural elders (primarily improving functional health). The heterogeneity results align with these predictions, thus linking mechanism evidence with equity-oriented policy implications, which is a dimension rarely addressed in prior research.

## Policy Background, theoretical Framework, and research hypotheses

### Policy background

In order to coordinate and integrate home-based, community-based, and institutional elderly care models and establish a multi-tiered elderly care service system, the Ministry of Civil Affairs and the Ministry of Finance of China jointly issued the Notice on the Central Government Financial Support for Carrying out Reforms in Home-based and Community Elderly Care Services Pilot Programs in 2016 to launch pilot reforms in home-based and community elderly care services. From 2016 to 2020, the pilot program engaged five batches of 203 cities nationwide. The central government invested 1 billion yuan annually, totaling about 5 billion yuan, through the central special lottery public welfare fund to encourage local governments to increase policy innovation and capital investment, which has a capital leverage effect. During the pilot period, the local government invested more than 18 billion yuan, while social investment reached at least 13 billion yuan, resulting in a total investment scale exceeding 36 billion yuan. The original intention of small investment by the central government and large investment by local governments and private capital was realized.

In terms of policy content, the pilots constitute a comprehensive package centered on improving elderly care accessibility. First, infrastructure and age-friendly retrofitting lowered barriers through subsidies tied to product prices and through new community facilities—for example, Yingkou in Liaoning province used RMB 28.17 million in central pilot funds plus RMB 81.39 million in local matching to build 40 urban–rural community eldercare centers and renovate 12 community sites, equipping them with safety monitoring and day-care functions. Second, smart eldercare platforms harness big data and connected devices for beneficiary identification, needs assessment, service dispatch, and quality oversight—for instance, Guangxi province Beihai’s smart eldercare data center registered information on 100,000 older adults and distributed smoke detectors, smart wristbands, and similar devices to vulnerable groups to reduce risks of wandering and accidents. Third, operational innovation and subsidies align “government leadership + market operation” through purchasing and performance mechanisms, yielding networked facilities and standardized service delivery. Hubei province Wuhan’s three-tier model of “community-embedded + hub-and-spoke + combined centralized–decentralized governance” integrates more than 1,800 offline providers to deliver on-demand meals, bathing, and medical assistance, with construction subsidies for service points (up to RMB 500,000 for community-embedded sites and RMB 1,000,000 for hub sites) and caregiver position subsidies (RMB 100 per person per month). Fourth, protection for priority groups provides heavy-disabled, disabled, and low-income moderately disabled older adults with care equipment and monitoring devices, connects them to government oversight platforms, and ensures follow-up services, thereby addressing equity and affordability.

In terms of actors, at the center, the Ministry of Civil Affairs (MCA) and the Ministry of Finance (MOF) set top-level objectives and funding rules for the HCBS pilots (five batches, 203 localities since 2016), while mandating province-level summary and diffusion of local experience. Local government leadership forges vertical linkage and horizontal coordination (many pilot localities set up mayor or district head–led task forces, while streets/sub-districts and communities execute facility roll-out and frontline provision. Market actors expand supply via socialized operation and capital leverage, using “public construction + private operation” and “private operation with public support” to bring in professional providers. Social forces fill gaps left by informal care: the Hubei province Huangshi Wufu Volunteer Association delivered some 74,000 instances of meal assistance; Anqing incubated 23 social organizations to deliver publicly funded projects.

The policy process operates as a closed loop. Agenda-setting is pain-point driven and data-led. Policy design combines rule-making with tool innovation, with smart oversight and payment reform. Implementation blends resource integration with model exploration, and supervision/evaluation couples technology with performance. Overall, government, market, society, and families co-evolve around the goals of accessibility, affordability, quality, continuity, and equity. Principal–agent and incentive–constraint mechanisms are continually recalibrated across actors, propelling the pilots along a trajectory of “lowering access barriers—strengthening supply capacity—reinforcing the service closed loop.”

In fact, according to the implementation pathways of HCBS, the overall policy outlines a systemic reform focused on “enhancing the accessibility of elderly care services” as its core goal, developing along the trajectory of “lowering access barriers—consolidating supply capacity—strengthening the service continuum. The common aim of the pilots is not only to shift from “whether services exist” to “whether they are accessible, affordable, and effective,” but also to internalize service accessibility into comprehensive improvements in affordability, quality, continuity, and equity.

The five batches of pilot cities were launched at different times: 26 cities in November 2016, 50 cities in November 2017, 71 cities in May 2018, 54 cities in August 2019, and 59 cities in February 2020. Considering that CHARLS surveys are typically conducted in summer, and that the policy effects take time to materialize. The 2018 and 2020 pilots would not immediately influence older adults’ health outcomes. Therefore, in this study, the fifth batch of pilot cities is treated as being exposed in 2021. As a result, the treatment group in the data includes the first four batches of pilot cities, while the control group consists of the fifth batch and the non-pilot cities.

### Theoretical framework and research hypotheses

Within Grossman’s (1972) health demand model, elderly health is conceptualized as ‘health capital’ (H). This capital stock depreciates over time but can be augmented through health investments. Individuals produce health capital by allocating time and market goods, while eldercare policies alter seniors’ investment levels by influencing the price and accessibility of health services, thereby determining health outcomes.

According to the Health Capital-Health Production framework, elderly health capital$\:{\mathrm{H}}_{\mathrm{i}\mathrm{t}}$ is jointly determined by inputs of medical/eldercare services$\:{\mathrm{m}}_{\mathrm{i}\mathrm{t}}$, time allocation$\:{\mathrm{t}}_{\mathrm{i}\mathrm{t}}$, individual endowments$\:{\mathrm{E}}_{\mathrm{i}}$, age-related factors$\:{\mathrm{A}}_{\mathrm{i}\mathrm{t}}$, and accessibility of care services$\:{\mathrm{S}}_{\mathrm{i}\mathrm{t}}$.

$$\:{\mathrm{H}}_{\mathrm{i}\mathrm{t}}=\mathrm{H}({\mathrm{m}}_{\mathrm{i}\mathrm{t}},{\mathrm{t}}_{\mathrm{i}\mathrm{t}},{\mathrm{E}}_{\mathrm{i}},{\mathrm{A}}_{\mathrm{i}\mathrm{t}},{\mathrm{S}}_{\mathrm{i}\mathrm{t}};{\uptheta\:})$$

Health capital depreciates over time but is net-augmented through health investments$\:{\mathrm{I}}_{\mathrm{i}\mathrm{t}}$:

$$\:{\mathrm{H}}_{\mathrm{i},\mathrm{t}+1}=(1-{\updelta\:}){\mathrm{H}}_{\mathrm{i}\mathrm{t}}+{\mathrm{I}}_{\mathrm{i}\mathrm{t}}$$

Health investment is modeled in a testable Cobb-Douglas functional form:

$$\:{\mathrm{I}}_{\mathrm{i}\mathrm{t}}={\upalpha\:}{\mathrm{m}}_{\mathrm{i}\mathrm{t}}^{{\upbeta\:}1}{\mathrm{t}}_{\mathrm{i}\mathrm{t}}^{{\upbeta\:}2}{\mathrm{S}}_{\mathrm{i}\mathrm{t}}^{{\upgamma\:}}({\upgamma\:}>0)\:\:\:\:\:\:\:\:\:\:\:\:\:\:\:\:\:\:\:\:\:\:\:\:\:\:\:\:\:\:\:$$

In this model, $\:{\mathrm{S}}_{\mathrm{i}\mathrm{t}\:}$denotes the accessibility of elderly care services, while α, β, and γ represent technical parameters.

Individuals confront both budgetary and temporal constraints:

$$\:{\mathrm{Y}}_{\mathrm{i}}={\mathrm{p}}_{\mathrm{m},\mathrm{i}\mathrm{t}}{\mathrm{m}}_{\mathrm{i}\mathrm{t}}+{{\upomega\:}}_{\mathrm{i}}(\mathrm{T}-{\mathrm{t}}_{\mathrm{i}\mathrm{t}})+{\mathrm{C}}_{\mathrm{i}\mathrm{t}}$$

$\:{\mathrm{Y}}_{\mathrm{i}}$denotes income, $\:{\mathrm{p}}_{\mathrm{m},\mathrm{i}\mathrm{t}}$represents the price of medical services, $\:{{\upomega\:}}_{\mathrm{i}}$ is the opportunity cost of time, $\:\mathrm{T}$ indicates total available time, and $\:{\mathrm{C}}_{\mathrm{i}\mathrm{t}}$signifies other consumption expenditures.

$$\:\mathrm{T}={\mathrm{t}}_{\mathrm{i}\mathrm{t}}+{\mathrm{l}}_{\mathrm{i}\mathrm{t}}+{\mathrm{s}}_{\mathrm{i}\mathrm{t}}$$

$\:{\mathrm{l}}_{\mathrm{i}\mathrm{t}}$ denotes labor hours (retired elderly is 0), and $\:{\mathrm{s}}_{\mathrm{i}\mathrm{t}}$ captures time allocated to other activities.

In order to embed policy into the model, we assume the accessibility of elderly care service is exogenously enhanced by policy interventions.

$$\:{\mathrm{S}}_{\mathrm{i}\mathrm{t}}={\mathrm{S}}_{0}+{\Delta\:}{\mathrm{S}}^{\mathrm{p}\mathrm{o}\mathrm{l}\mathrm{i}\mathrm{c}\mathrm{y}}{\mathrm{p}\mathrm{o}\mathrm{l}\mathrm{i}\mathrm{c}\mathrm{y}}_{\mathrm{i}\mathrm{t}}$$

$\:{\mathrm{S}}_{0}$denotes the baseline accessibility level prior to policy implementation, $\:{\Delta\:}{\mathrm{S}}^{\mathrm{p}\mathrm{o}\mathrm{l}\mathrm{i}\mathrm{c}\mathrm{y}}$represents the policy-induced improvement in accessibility, and $\:{\mathrm{p}\mathrm{o}\mathrm{l}\mathrm{i}\mathrm{c}\mathrm{y}}_{\mathrm{i}\mathrm{t}}$ is a binary indicator variable (coded as 1 for policy-implemented periods, and 0 otherwise).

Enhancements in elderly care service accessibility are primarily achieved through reductions in three categories of costs: information costs, time costs, and economic costs.

$$\:{\mathrm{I}\mathrm{C}}_{\mathrm{i}\mathrm{t}}={\mathrm{I}\mathrm{C}}_{0}{\mathrm{e}}^{-{{\upvarphi\:}}_{1}{\mathrm{S}}_{\mathrm{i}\mathrm{t}}},{\mathrm{T}\mathrm{C}}_{\mathrm{i}\mathrm{t}}={\mathrm{T}\mathrm{C}}_{0}{\mathrm{e}}^{-{{\upvarphi\:}}_{2}{\mathrm{S}}_{\mathrm{i}\mathrm{t}}},{\mathrm{E}\mathrm{C}}_{\mathrm{i}\mathrm{t}}={\mathrm{E}\mathrm{C}}_{0}{\mathrm{e}}^{-{{\upvarphi\:}}_{3}{\mathrm{S}}_{\mathrm{i}\mathrm{t}}}$$

$\:{{\upvarphi\:}}_{1}$, $\:{{\upvarphi\:}}_{2}$,and $\:{{\upvarphi\:}}_{3}$ (all greater than zero) denote elasticity parameters.

the effective price of eldercare services:

$$\:{\mathrm{p}}_{\mathrm{m},\mathrm{i}\mathrm{t}}^{\mathrm{e}\mathrm{f}\mathrm{f}\mathrm{e}\mathrm{c}\mathrm{t}\mathrm{i}\mathrm{v}\mathrm{e}}={\mathrm{p}}_{\mathrm{m},\mathrm{i}\mathrm{t}}^{\mathrm{n}\mathrm{o}\mathrm{m}\mathrm{i}\mathrm{n}\mathrm{a}\mathrm{l}}+{\mathrm{I}\mathrm{C}}_{\mathrm{i}\mathrm{t}}+{\mathrm{T}\mathrm{C}}_{\mathrm{i}\mathrm{t}}+{\mathrm{E}\mathrm{C}}_{\mathrm{i}\mathrm{t}}$$

After implmentation of the HCBS:

$$\:{\mathrm{p}}_{\mathrm{m},\mathrm{i}\mathrm{t}}^{\mathrm{e}\mathrm{f}\mathrm{f}\mathrm{e}\mathrm{c}\mathrm{t}\mathrm{i}\mathrm{v}\mathrm{e}}={\mathrm{p}}_{\mathrm{m},\mathrm{i}\mathrm{t}}^{\mathrm{n}\mathrm{o}\mathrm{m}\mathrm{i}\mathrm{n}\mathrm{a}\mathrm{l}}+{\mathrm{I}\mathrm{C}}_{0}{\mathrm{e}}^{-{{\upvarphi\:}}_{1}{\mathrm{S}}_{\mathrm{i}\mathrm{t}}}+{\mathrm{T}\mathrm{C}}_{0}{\mathrm{e}}^{-{{\upvarphi\:}}_{2}{\mathrm{S}}_{\mathrm{i}\mathrm{t}}}+{\mathrm{E}\mathrm{C}}_{0}{\mathrm{e}}^{-{{\upvarphi\:}}_{3}{\mathrm{S}}_{\mathrm{i}\mathrm{t}}}$$

The price of eldercare services$\:{\:\mathrm{p}}_{\mathrm{m},\mathrm{i}\mathrm{t}}^{\mathrm{e}\mathrm{f}\mathrm{f}\mathrm{e}\mathrm{c}\mathrm{t}\mathrm{i}\mathrm{v}\mathrm{e}}$ decreases due to increase about the accessibility of elderly care service $\:{\mathrm{S}}_{\mathrm{i}\mathrm{t}}$.

Elderly lifetime utility maximization:

$$\:{\mathrm{m}\mathrm{a}\mathrm{x}}_{{\mathrm{m}}_{\mathrm{i}\mathrm{t}}{,\mathrm{t}}_{\mathrm{i}\mathrm{t}}}\mathrm{U}=\sum\:_{\mathrm{t}=60}^{{\mathrm{T}}_{\mathrm{m}\mathrm{a}\mathrm{x}}}{{\upbeta\:}}^{\mathrm{t}-60}{\upmu\:}\left({\mathrm{H}}_{\mathrm{i}\mathrm{t}}, {\mathrm{C}}_{\mathrm{i}\mathrm{t}}\right)$$

Incorporating both budget and time constraints into the individual maximization problem and constructing the Lagrangian function yield the first-order conditions for optimal resource allocation.

$$\:\frac{{\updelta\:}{\upmu\:}}{{\updelta\:}{\mathrm{H}}_{\mathrm{i}\mathrm{t}}}\frac{{\updelta\:}{\mathrm{H}}_{\mathrm{i}\mathrm{t}}}{{\updelta\:}{\mathrm{I}}_{\mathrm{i}\mathrm{t}}}\frac{{\updelta\:}{\mathrm{I}}_{\mathrm{i}\mathrm{t}}}{{\updelta\:}{\mathrm{m}}_{\mathrm{i}\mathrm{t}}}={\uplambda\:}{\mathrm{p}}_{\mathrm{m},\mathrm{i}\mathrm{t}}^{\mathrm{e}\mathrm{f}\mathrm{f}\mathrm{e}\mathrm{c}\mathrm{t}\mathrm{i}\mathrm{v}\mathrm{e}},\:\frac{{\updelta\:}{\upmu\:}}{{\updelta\:}{\mathrm{H}}_{\mathrm{i}\mathrm{t}}}\frac{{\updelta\:}{\mathrm{H}}_{\mathrm{i}\mathrm{t}}}{{\updelta\:}{\mathrm{I}}_{\mathrm{i}\mathrm{t}}}\frac{{\updelta\:}{\mathrm{I}}_{\mathrm{i}\mathrm{t}}}{{\updelta\:}{\mathrm{t}}_{\mathrm{i}\mathrm{t}}}={\uplambda\:}{{\upomega\:}}_{\mathrm{i}}$$

The first-order conditions characterize the optimality conditions for elderly care inputs $\:{\mathrm{m}}_{\mathrm{i}\mathrm{t}}$and time inputs $\:{\mathrm{t}}_{\mathrm{i}\mathrm{t}}$, and equilibrium between Marginal Utility Gain and Resource Shadow Price. The comparative statics reveal that when policy-enhanced accessibility $\:{\mathrm{S}}_{\mathrm{i}\mathrm{t}}$reduces effective prices and time opportunity costs, optimal inputs $\:{\mathrm{m}}_{\mathrm{i}\mathrm{t}}$and $\:{\mathrm{t}}_{\mathrm{i}\mathrm{t}}$ increase, thereby elevating health investment $\:{\mathrm{I}}_{\mathrm{i}\mathrm{t}}$ and health capital $\:{\mathrm{H}}_{\mathrm{i}\mathrm{t}}$.

Therefore, Hypothesis 1: The HCBS significantly improves elderly health outcomes, evidenced by reduced depressive symptoms (measured by CESD scale) and diminished functional limitations in daily living (assessed by ADL index).

Accordingly, policy effects can be decomposed into “direct effects” and three types of “indirect effects”.​.

The policy enhances the accessibility of elderly care services $\:{\mathrm{S}}_{\mathrm{i}\mathrm{t}}$, which directly increases health investment $\:{\mathrm{I}}_{\mathrm{i}\mathrm{t}}$. From the health investment function:

$$\:{\mathrm{I}}_{\mathrm{i}\mathrm{t}}={\upalpha\:}{\mathrm{m}}_{\mathrm{i}\mathrm{t}}^{{{\upbeta\:}}_{1}}{\mathrm{t}}_{\mathrm{i}\mathrm{t}}^{{{\upbeta\:}}_{2}}{\mathrm{s}}_{\mathrm{i}\mathrm{t}}^{{\upgamma\:}},{\upgamma\:}>0$$

Taking the partial derivative with respect to $\:{\mathrm{S}}_{\mathrm{i}\mathrm{t}}$ yields,

$$\:\frac{{\updelta\:}{\mathrm{I}}_{\mathrm{i}\mathrm{t}}}{{\updelta\:}{\mathrm{S}}_{\mathrm{i}\mathrm{t}}}={\upgamma\:}{\upalpha\:}{\mathrm{m}}_{\mathrm{i}\mathrm{t}}^{{{\upbeta\:}}_{1}}{\mathrm{t}}_{\mathrm{i}\mathrm{t}}^{{{\upbeta\:}}_{2}}{\mathrm{s}}_{\mathrm{i}\mathrm{t}}^{{\upgamma\:}-1}>0$$

Substituting this into the health capital evolution equation:​

$$\begin{aligned} \:\frac{{\updelta\:}{\mathrm{H}}_{\mathrm{i},\mathrm{t}+1}}{{\updelta\:}{\mathrm{p}\mathrm{o}\mathrm{l}\mathrm{i}\mathrm{c}\mathrm{y}}_{\mathrm{i}\mathrm{t}}}=\frac{{\updelta\:}{\mathrm{H}}_{\mathrm{i},\mathrm{t}+1}}{{\updelta\:}{\mathrm{I}}_{\mathrm{i}\mathrm{t}}}\cdot\frac{{\updelta\:}{\mathrm{I}}_{\mathrm{i}\mathrm{t}}}{{\updelta\:}{\mathrm{S}}_{\mathrm{i}\mathrm{t}}}\cdot\frac{{\updelta\:}{\mathrm{S}}_{\mathrm{i}\mathrm{t}}}{{\updelta\:}{\mathrm{p}\mathrm{o}\mathrm{l}\mathrm{i}\mathrm{c}\mathrm{y}}_{\mathrm{i}\mathrm{t}}}>0 \end{aligned}$$.

The direct effect indicates that the HCBS directly increases health investment and thereby improves health outcomes by enhancing accessibility $\:{\mathrm{S}}_{\mathrm{i}\mathrm{t}}$, without operating through the channels of price, time, or information.

By enhancing the accessibility of elderly care services $\:{\mathrm{S}}_{\mathrm{i}\mathrm{t}}$, the HCBS indirectly affects older adults’ health investments $\:{\mathrm{I}}_{\mathrm{i}\mathrm{t}}$ and depend on three channels: a price effect, a time effect, and an information effect.

Improved accessibility lowers the effective price of elderly care services:

$$\:\frac{{\updelta\:}{\mathrm{p}}_{\mathrm{m},\mathrm{i}\mathrm{t}}^{\mathrm{e}\mathrm{f}\mathrm{f}\mathrm{e}\mathrm{c}\mathrm{t}\mathrm{i}\mathrm{v}\mathrm{e}}}{{\updelta\:}{\mathrm{S}}_{\mathrm{i}\mathrm{t}}}<0$$

increasing demand for elderly care services and health investment:

$$\begin{aligned} \:\frac{{\updelta\:}{\mathrm{I}}_{\mathrm{i}\mathrm{t}}}{{\updelta\:}{\mathrm{S}}_{\mathrm{i}\mathrm{t}}}=\frac{{\updelta\:}{\mathrm{I}}_{\mathrm{i}\mathrm{t}}}{{\updelta\:}{\mathrm{p}}_{\mathrm{m},\mathrm{i}\mathrm{t}}^{\mathrm{e}\mathrm{f}\mathrm{f}\mathrm{e}\mathrm{c}\mathrm{t}\mathrm{i}\mathrm{v}\mathrm{e}}}\cdot\frac{{\updelta\:}{\mathrm{p}}_{\mathrm{m},\mathrm{i}\mathrm{t}}^{\mathrm{e}\mathrm{f}\mathrm{f}\mathrm{e}\mathrm{c}\mathrm{t}\mathrm{i}\mathrm{v}\mathrm{e}}}{{\updelta\:}{\mathrm{S}}_{\mathrm{i}\mathrm{t}}}>0 \end{aligned}$$

Enhanced accessibility lowers the time cost of accessing services:

$$\:\frac{{\updelta\:}{\mathrm{T}\mathrm{C}}_{\mathrm{i}\mathrm{t}}}{{\updelta\:}{\mathrm{S}}_{\mathrm{i}\mathrm{t}}}<0$$

The freed-up time can be reallocated to health investment:

$$\begin{aligned} \:\frac{{\updelta\:}{\mathrm{I}}_{\mathrm{i}\mathrm{t}}}{{\updelta\:}{\mathrm{S}}_{\mathrm{i}\mathrm{t}}}=\frac{{\updelta\:}{\mathrm{I}}_{\mathrm{i}\mathrm{t}}}{{\updelta\:}{\mathrm{T}\mathrm{C}}_{\mathrm{i}\mathrm{t}}}\cdot\frac{{\updelta\:}{\mathrm{T}\mathrm{C}}_{\mathrm{i}\mathrm{t}}}{{\updelta\:}{\mathrm{S}}_{\mathrm{i}\mathrm{t}}}>0 \end{aligned}$$

Improved accessibility reduces information costs:

$$\:\frac{{\updelta\:}{\mathrm{I}\mathrm{C}}_{\mathrm{i}\mathrm{t}}}{{\updelta\:}{\mathrm{S}}_{\mathrm{i}\mathrm{t}}}<0$$

thereby increasing the efficiency of investment:

$$\begin{aligned} \:\frac{{\updelta\:}{\mathrm{I}}_{\mathrm{i}\mathrm{t}}}{{\updelta\:}{\mathrm{S}}_{\mathrm{i}\mathrm{t}}}=\frac{{\updelta\:}{\mathrm{I}}_{\mathrm{i}\mathrm{t}}}{{\updelta\:}{\mathrm{I}\mathrm{C}}_{\mathrm{i}\mathrm{t}}}\cdot\frac{{\updelta\:}{\mathrm{I}\mathrm{C}}_{\mathrm{i}\mathrm{t}}}{{\updelta\:}{\mathrm{S}}_{\mathrm{i}\mathrm{t}}}>0 \end{aligned}$$

Combining the direct and indirect effects, the total effect can be expressed as:

$$\:\frac{{\updelta\:}{\mathrm{H}}_{\mathrm{i}\mathrm{t}}}{{\updelta\:}{\mathrm{p}\mathrm{o}\mathrm{l}\mathrm{i}\mathrm{c}\mathrm{y}}_{\mathrm{i}\mathrm{t}}}=\frac{{\updelta\:}{\mathrm{H}}_{\mathrm{i}\mathrm{t}}}{{\updelta\:}{\mathrm{I}}_{\mathrm{i}\mathrm{t}}}\cdot\left(\frac{{\updelta\:}{\mathrm{I}}_{\mathrm{i}\mathrm{t}}}{{\updelta\:}{\mathrm{S}}_{\mathrm{i}\mathrm{t}}}\right)\cdot\frac{{\updelta\:}{\mathrm{S}}_{\mathrm{i}\mathrm{t}}}{{\updelta\:}{\mathrm{p}\mathrm{o}\mathrm{l}\mathrm{i}\mathrm{c}\mathrm{y}}_{\mathrm{i}\mathrm{t}}}$$

$$\begin{aligned} \:\frac{{\updelta\:}{\mathrm{H}}_{\mathrm{i}\mathrm{t}}}{{\updelta\:}{\mathrm{p}\mathrm{o}\mathrm{l}\mathrm{i}\mathrm{c}\mathrm{y}}_{\mathrm{i}\mathrm{t}}}=&\frac{{\updelta\:}{\mathrm{H}}_{\mathrm{i}\mathrm{t}}}{{\updelta\:}{\mathrm{I}}_{\mathrm{i}\mathrm{t}}}\cdot\left({\upgamma\:}{\upalpha\:}{\mathrm{m}}_{\mathrm{i}\mathrm{t}}^{{{\upbeta\:}}_{1}}{\mathrm{t}}_{\mathrm{i}\mathrm{t}}^{{{\upbeta\:}}_{2}}{\mathrm{s}}_{\mathrm{i}\mathrm{t}}^{{\upgamma\:}-1}+\frac{{\updelta\:}{\mathrm{I}}_{\mathrm{i}\mathrm{t}}}{{\updelta\:}{\mathrm{p}}_{\mathrm{m},\mathrm{i}\mathrm{t}}^{\mathrm{e}\mathrm{f}\mathrm{f}\mathrm{e}\mathrm{c}\mathrm{t}\mathrm{i}\mathrm{v}\mathrm{e}}}\right. \\& \left.\cdot\frac{{\updelta\:}{\mathrm{p}}_{\mathrm{m},\mathrm{i}\mathrm{t}}^{\mathrm{e}\mathrm{f}\mathrm{f}\mathrm{e}\mathrm{c}\mathrm{t}\mathrm{i}\mathrm{v}\mathrm{e}}}{{\updelta\:}{\mathrm{S}}_{\mathrm{i}\mathrm{t}}}+\frac{{\updelta\:}{\mathrm{I}}_{\mathrm{i}\mathrm{t}}}{{\updelta\:}{\mathrm{T}\mathrm{C}}_{\mathrm{i}\mathrm{t}}} \cdot\frac{{\updelta\:}{\mathrm{T}\mathrm{C}}_{\mathrm{i}\mathrm{t}}}{{\updelta\:}{\mathrm{S}}_{\mathrm{i}\mathrm{t}}}+\frac{{\updelta\:}{\mathrm{I}}_{\mathrm{i}\mathrm{t}}}{{\updelta\:}{\mathrm{I}\mathrm{C}}_{\mathrm{i}\mathrm{t}}}\cdot\frac{{\updelta\:}{\mathrm{I}\mathrm{C}}_{\mathrm{i}\mathrm{t}}}{{\updelta\:}{\mathrm{S}}_{\mathrm{i}\mathrm{t}}}\right)\\& \cdot\frac{{\updelta\:}{\mathrm{S}}_{\mathrm{i}\mathrm{t}}}{{\updelta\:}{\mathrm{p}\mathrm{o}\mathrm{l}\mathrm{i}\mathrm{c}\mathrm{y}}_{\mathrm{i}\mathrm{t}}} \end{aligned}$$

Hypothesis 2: The HCBS increases health investment and improves health outcomes by raising the accessibility of elderly care services,

Based on the systematic differences in shadow prices and marginal benefits across different groups, health improvement policies exhibit predictable heterogeneity. Psychosocial pathway dominates among advantaged groups. The elderly with higher education and without chronic diseases show greater sensitivity to reduced information costs. This enables more effective alleviation of psychological uncertainty through information channels, leading to concentrated improvements in CESD scores. Besides, the Physical pathway dominates among vulnerable groups. Rural elderly with chronic diseases, lower education, and higher baseline functional limitations face greater time/economic constraints. Policy-driven accessibility enhancements and subsidies generate higher marginal productivity of health investments for these groups, resulting in concentrated improvements in ADL.

Thus, Hypothesis 3: the HCBS improve mental health among chronic disease-free and highly-educated individuals, while enhancing physical functioning among chronic conditions, lower education, and rural elders.

## Data and methodology

### Data

The data for this study were derived from the China Health and Retirement Longitudinal Survey (CHARLS) [17], spanning the years 2011 to 2020. The baseline survey, initiated in 2011, covers residents aged 45 and older across counties, towns, and villages in 28 provinces nationwide. The dataset includes comprehensive information on demographic characteristics, health status and functional capacity, healthcare utilization and insurance coverage, income, expenditures, and assets. In addition, the CHARLS survey constructs a nationally representative sample through the following steps. First, it covers 28 provincial-level administrative regions in China, ensuring representation of county/district-level differences across eastern, central, and western regions. Second, approximately 150 counties or districts were selected nationwide from 28 provinces using a multi-stage stratified sampling method, where the sampling frame was stratified by the urban–rural distribution (approximately 80.5% rural and 19.5% urban) to ensure representativeness. Third, within each county, three administrative villages or communities are randomly chosen, yielding about 450 primary sampling units in total. At the village/community level, households with members aged 45 years or above are randomly sampled from the household registration list, with around 20–30 households per unit. Finally, in each household, one individual aged 45 or older is randomly selected as the core respondent, with their spouse also eligible to participate regardless of age. The database only provides data at the provincial and municipal levels, with no information below the municipal level. CHARLS data are recognized for their representativeness of the aging population in China, methodological rigor, and longitudinal stability. In addition, the home and community-based elderly care service reform pilot program was primarily implemented during 2016–2020. The CHARLS 2011–2020 dataset, covering the period before and after the policy intervention, maintains consistent survey methodology and sampling frameworks. Although the fifth batch of pilot cities was announced in February 2020, the delayed allocation of fiscal funds and full implementation of services introduced temporal confounding effects. In order to address it, we excluded the fifth batch from the treatment group and retained only the first four batches (2016–2019) for policy effect analysis. After removing outliers and samples with missing covariates, the final analytical cohort comprised 25,287 respondents aged 60 years or older.

### Variable description

The key explanatory variable in this study is defined as “whether elderly residents live in a pilot region implementing the home and community-based elderly care service reform,” coded as 1 for areas where the reform was implemented and 0 otherwise. The CHARLS database covers 51 pilot regions from the first four batches of this nationwide reform pilot.

The explained variable was the health level of the elderly. This paper measures the effect of the pilot reform of home and community-based elderly care service on the health level of the elderly from two dimensions of functional health and mental health. Firstly, functional health is represented by Activities of Daily Living (ADL). The assessment of this variable integrated two dimensions: Basic Activities of Daily Living (BADL) and Instrumental Activities of Daily Living (IADL). BADL encompasses six activities: getting in and out of bed, dressing, eating, using the toilet, transferring, and bathing. IADL includes five key activities: preparing meals, cleaning/housekeeping, shopping, managing money, and taking medications. A composite scale was constructed by summing the scores from the 6 BADL items and the 5 IADL items, resulting in a total score ranging from 0 to 11. Respondents received a score of 0 for no difficulty in any ADL item, 1 point for having difficulty in one item, 2 points for having difficulty in two items, up to a maximum of 11 points. Within this scoring system, a higher total score indicates that the respondent experiences more difficulties in daily life, signifying poorer functional health status and a greater dependence on others for care. Combining BADL and IADL into a single composite indicator provides a more comprehensive and continuous measure of the overall functional status of older adults, ranging from basic self-care to complex instrumental activities [18, 19]. This comprehensive assessment method helps to more accurately quantify their level of health impairment and the corresponding need for support. In addition, the measurement of mental health in this study utilized the “Epidemiological Studies Depression Scale” (CESD) scores from the CHARLS. This scale focuses on individuals’ emotional experiences and comprises: (1) eight negative emotional items assessing whether older adults experienced being bothered by trivial matters, difficulty concentrating, feeling depressed, effortful task completion, experiencing fear, poor sleep quality, loneliness, and inability to continue daily life; (2) two positive emotional items evaluating hopefulness about the future and feelings of happiness. All 10 items employed a 4-point scoring system (0 = rarely or none, 1 = occasionally, 2 = sometimes, 3 = most of the time), with reverse scoring applied to the two positive items. A comprehensive depression index was constructed by summing the scores across all 10 items, yielding a total score ranging from 0 to 30. Higher CESD scores indicate poorer mental health status in the elderly.

Control Variables. Based on Grossman’s (1972) health demand model, and to reduce potential confounding factors in the policy estimation, this paper introduces four categories of control variables: individual endowment, socioeconomic status, lifestyle habits, and health conditions. Individual endowments include age and marital status. Socioeconomic conditions include household registration, retirement status, household consumption level, education level, and the number of surviving children, aiming to capture differences in resource accessibility, health investment capacity, and social capital. Specifically, controlling for household registration helps isolate inherent urban-rural differences, such as those in the dual urban-rural structure and disparities in pension resources, thereby allowing for a more accurate identification of the policy’s net effect [20, 21]. Per capita consumption (log-transformed) helps eliminate interference from economic factors, avoiding overestimation or underestimation of the policy’s impact on health outcomes [22]. Lifestyle habits include smoking status and nap time to isolate the disturbance of key health behaviors. Health conditions include chronic diseases, hospitalization experience in the past year, and participation in medical insurance. All regression models include individual and year fixed effects, and standard errors are clustered at the individual level. Variable definitions and descriptive statistics are presented in the accompanying Table 1.

**Table 1:** Descriptive statistics

Variable	Mean	Rural	City
CESD score	8.737	9.373	6.779
ADL score	1.113	1.202	0.792
Age	4.217	4.216	4.226
Retirement	0.212	0.438	0.733
Marital Status	0.821	0.809	0.841
Household registration	0.751	1	0
Smoke	0.462	0.457	0.468
Household consumption	8.954	8.847	9.518
Number of surviving children	3.083	3.214	2.507
Nap time	0.521	0.524	0.591
Hospitalizations or not	0.219	0.339	0.394
Medical insurance	0.046	0.008	0.158
Physical examination	0.976	0.976	0.990
Education	1.821	1.578	2.544
Chronic	0.827	0.819	0.851

### Model specification

Since the pilot reform of home and community-based elderly care services was implemented in five staggered batches, this study adopts a staggered difference-in-differences (DID) framework to evaluate the policy effects. The baseline regression model is specified as follows:

$$\:{Health}_{ict}={\beta\:}_{0}+{\beta\:}_{1}{Treat}_{ic}\times\:{Post}_{t}+\gamma\:{X}_{ict}+{\mu\:}_{c}+{\mu\:}_{t}+{\mu\:}_{i}+{\xi\:}_{ict},$$

Subscripts i, c and t represent individual, city and year dimensions respectively. $\:{\mathrm{H}\mathrm{e}\mathrm{a}\mathrm{l}\mathrm{t}\mathrm{h}}_{\mathrm{i}\mathrm{c}\mathrm{t}}$ indicates the health level of the elderly i living in city c in year t, including CESD score and ADL score. $\:{\mathrm{T}\mathrm{r}\mathrm{e}\mathrm{a}\mathrm{t}}_{\mathrm{i}\mathrm{c}}\times\:{\mathrm{P}\mathrm{o}\mathrm{s}\mathrm{t}}_{\mathrm{t}}$ represents the interaction term between the virtual variable of the policy and the virtual variable of the policy implementation time. If the elderly individual i is located in city c where is the policy implemented in year t, it represents that the individual enters the processing period and the value is 1, otherwise, the value is 0. $\:{{\upbeta\:}}_{1}$ is the most concerned coefficient to measure the effect of the pilot reform of home and community-based elderly care services. $\:{\mathrm{X}}_{\mathrm{i}\mathrm{c}\mathrm{t}}$ is the control variable about the individual characteristics, living habits and family status of the elderly in this paper, including age, retirement, marital status, household registration, smoking, per capita consumption level of the family, number of living children, etc. $\:{{\upmu\:}}_{\mathrm{c}}$ refers to urban fixed effect, which is used to control all factors that do not change with time at the city level.$\:\:{{\upmu\:}}_{\mathrm{t}}$ indicates the year fixed effect, used to control the characteristics of the time level that do not change with the region. $\:{{\upmu\:}}_{\mathrm{i}}$ is the individual fixed effect, used to control all the factors that do not change with time.$\:\:{{\upxi\:}}_{\mathrm{i}\mathrm{c}\mathrm{t}}$ is the random disturbance term.

## Empirical results

### Regression analysis

The regression results on the impact of the HCBS on elderly health outcomes are presented in the Table 2. Columns (1) and (2) report the baseline results for the CESD score and ADL score, respectively. Overall, the HCBS significantly increased the accessibility of elderly care services and improved health outcomes among older adults.

**Table 2:** Baseline regression results

Variables	(1) CESD score	(2) ADL score
* Treat*_ic_ x Post_t_	-0.3957^b^	-0.2237^c^
	(-2.0612)	(-3.8908)
Age	18.5728	-24.4711^c^
	(1.4761)	(-5.4531)
Retirement	0.2438	-0.0129
	(0.7405)	(-0.1591)
Marital Status	0.8032^b^	-0.0006
	(2.5220)	(-0.0070)
Household registration	0.0066	0.0153
	(0.0250)	(0.1556)
Smoke	0.1246^b^	0.0819^c^
	(2.2173)	(4.4735)
Household consumption	0.0627	-0.0354
	(0.7372)	(-1.2845)
Number of surviving children	-0.9714^c^	-0.1446^a^
	(-3.5684)	(-1.7090)
Nap time	0.7943^c^	0.3337^c^
	(6.4934)	(7.6305)
Number of hospitalizations	-0.1238	-0.0060
	(-1.2377)	(-0.1813)
Medical insurance	-0.0545	-0.0047
	(-0.2213)	(-0.0655)
cons	-70.8042	103.7501^c^
	(-1.3345)	(5.4847)
Sample Size	25,287	25,728
Adjusted R^2^	0.5176	0.5273

Note: Standard error for clustering to the individual level in parentheses; ^*a^, **^b^, and ****^c^ represent statistically significant at the 10%, 5%, and 1% level, respectively

On the one hand, in the mental health dimension, the CESD score declined significantly (coefficient = -0.396), which translates into a reduction of about 1.3% points on the 30-point scale (equivalent to roughly 4.53% of the sample mean). On the other hand, in the functional health dimension, the ADL score decreased significantly (coefficient = -0.244, corresponding to a reduction of about 2.03% points on the 11-point scale (equivalent to nearly 20.1% of the sample mean). These results indicate that the pilot reform exerted a consistent and statistically significant positive effect on both mental and functional health of the elderly.

Although the average reductions in CESD and ADL scores (-0.396 and − 0.244, respectively) represent relatively small percentage changes on their respective scales, their practical significance should be evaluated from a broader public health and policy implementation perspective. First, at the population level, the China Community Newspaper, supervised by the Ministry of Civil Affairs, reported that more than 150 million older adults have benefited from HCBS pilot programs. Even a modest average reduction of 0.396 points and 0.244 points in depressive symptoms per individual translates into a substantial cumulative benefit—effectively alleviating mild psychological burdens across a massive population and preventing potential functional decline. This, in turn, reduces the demand for intensive care and healthcare expenditures, yielding significant social and economic benefits. Second, in light of the program’s scalability and sustainability, such small improvements should not be regarded as an endpoint but rather as the beginning of long-term positive impacts. The HCBS aims to establish a continuous support system for older adults, and these seemingly minor score improvements, if sustained over time, can slow health deterioration and ultimately result in meaningful differences in long-term health outcomes. Finally, it is important to note that the reported coefficients represent the average treatment effect. If interventions were targeted toward high-risk or unmet-need subgroups, such as rural or low-income older adults, the effect of HCBS on these populations could be considerably greater than the average. Therefore, a statistically significant and positive average effect already provides compelling evidence that HCBS constitutes a viable and valuable public policy for improving both the mental and functional health of older adults.

Within the DID framework, control variables are employed solely to purge confounding effects and are not intended for causal interpretation. The adjusted R² is approximately 0.52, indicating a reasonably good model fit and robust inference. Taken together, the results suggest that the pilot policy generated substantive and economically meaningful improvements in health across both mental and functional dimensions.

### Robustness

#### Parallel trend test

**Fig. 1:**
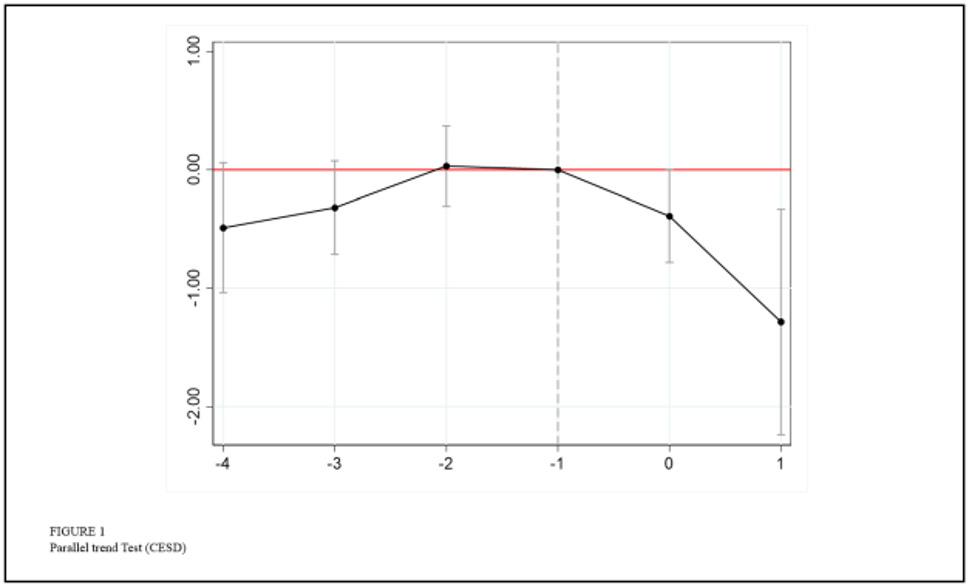
Parallel Test (CESD)

**Fig. 2:**
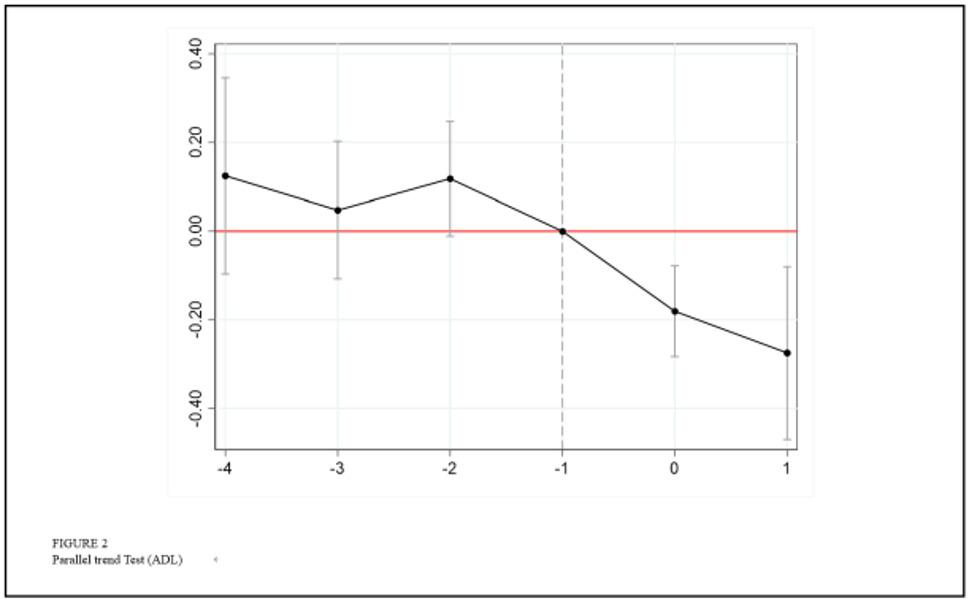
Parallel Test (ADL)

A critical assumption of the Staggered DID method is that the trends in the treatment and control groups should be the same before implementation of the policy. If the parallel trend assumption is violated, significant pre-existing trend differences in outcome variables between the treatment and control groups prior to policy implementation, the estimated results may be biased due to confounding trends. To address this methodological concern, we rigorously conducted parallel trend tests. The results demonstrated no statistically significant divergence in outcome variables between the two groups before the policy intervention, thereby supporting the robustness of our causal inference. Using the pre-policy period (Phase 1) as the baseline for individuals aged 60 and above, Figs. 1 and 2 illustrate the DID estimated coefficients with 90% confidence intervals respectively. The results indicate that all pre-treatment regression coefficients are statistically insignificant Fig. 1 for CESD scores and Fig. 2 for ADL scores). It demonstrates no divergent trends between treatment and control groups prior to the implementation of the pilot reform of home and community-based elderly care services. It satisfies the parallel trends assumption. After the implementation of the policy, the coefficients for the treatment group exhibit a statistically significant declining trend compared to the control group. This suggests that the pilot reform of home and community-based elderly care services generated progressively stronger positive effects on both mental health and functional health over time. Our analysis thus validates the parallel trends assumption, ensuring the credibility of causal estimates in this quasi-experimental design.

$$\:{Health}_{ict}={\beta\:}_{0}+\sum\:_{n=-3}^{2}{\beta\:}_{n}{Treat}_{ic}\times\:{Post}_{it}^{n}+\gamma\:{X}_{ict}+{\mu\:}_{c}+{\mu\:}_{t}+{\mu\:}_{i}+{\xi\:}_{ict}$$

#### Other robustness tests

The paper also conducted additional robustness tests, including a sensitivity analysis of parallel trends, Bacon decomposition analysis, an assessment of heterogeneous treatment effects, and a placebo test. The corresponding results and detailed analysis can be found in the Appendix [23–30]. Parallel trends sensitivity shows that Pre-treatment period event study coefficients show no statistically significant divergence between treated and control groups before policy implementation, supporting the parallel trends assumption. Further sensitivity checks leave the direction and significance of the core policy coefficients essentially unchanged. In addition, Bacon decomposition shows that, according to the decomposition of the two-way fixed effects (TWFE) estimator into its constituent 2 × 2 comparisons (e.g., early treated vs. never treated, late treated vs. earlier treated), following the Goodman-Bacon framework. The decomposition shows that over 96% of the information in our sample comes from the “clean” comparison of Treated vs. Never-Treated units. Besides, based on the heterogeneity-robust DID estimators and methodologies, this paper employs the dynamic effect tests of the event study approach [23]and the stacked event study estimation [24] Both sets of results corroborate the Bacon decomposition findings. Finally, Placebo tests show that these placebo regressions generally yield coefficients that are statistically indistinguishable from zero, reinforcing that the estimated effects in the actual specification are unlikely to be driven by spurious time trends or unobserved confounders Table 3

**Table 3:** Participation of the aged in the treatment and the control group in community elderly care services (%)

	service type	(1) Treatment mean	(2) Control mean	(3) Treatment-Control
Home and community aged care services	House visits	0.110	0.062	0.048^***a^
	Paid family doctors sign up for services	1.962	1.946	0.016***^a^

Note: *, **, and^***a^ represent statistically significant at the 10%, 5%, and 1% level, respectively

## Mechanism and heterogeneity

### Mechanism

The above research illustrates that the pilot reform of home and community elderly care services has had a significant positive impact on the functional health and mental health of the elderly. Based on the data of CHARLS in 2018, this paper calculated the accessibility of different types of community elderly care services for the elderly in the treatment group and the control group, and conducted a T-test for the difference between the groups. As shown in column (3) in Table 4, it reported the difference in participation rates between the treatment and control groups and the significance level of the T-test. Among them, the difference of home visit and paid family doctor contract service was significant, which indicates that the policy pilot effectively improved the accessibility of medical services for the elderly and improved the basic health management of the elderly. Due to the physical inconvenience caused by the aging of the elderly, the complexity of medical procedures, the difficulty of traveling to the hospital and the heavy medical burden, the elderly tend to reduce the number of medical visits or choose community health service centers. Moreover, there are still some problems in our country, such as unbalanced allocation of community health service resources, uneven connection of medical and old-age services, insufficient medical security funds, and an imperfect hierarchical diagnosis and treatment system. The HCBS improves the accessibility of medical services, helps release the medical service needs of the elderly, and protects the basic health rights of the elderly [31], which has a basic protective effect on the health of the elderly and can effectively improve the health of the elderly [32, 33]. According to the theoretical analysis above, the pilot reform of home and community elderly care services has an impact on the physical and mental health of the elderly through daily preventive health care, which will be further tested later.

**Table 4:** Mechanism results

	Physical examination
$\:{Treat}_{ic}\times\:{Post}_{t}$	0.0252^a^ (2.7420)
Control variables	Yes
Adjusted R^2^	0.2747

Note: Standard error for clustering to the individual level in parentheses; *, **, and ^a^ represent statistically significant at the 10%, 5%, and 1% level, respectively

The mechanism test results from Table 5 show that the Home- and Community-Based Elderly Care Reform pilot significantly increased the intensity of community-based care: the probability that older adults received routine preventive health care rose by 2.52% points (coefficient = 0.0252, *p* < 0.01). This indicates that the policy not only improved service accessibility but also enhanced the provision of community health management and preventive services, enabling older adults to more easily obtain ongoing health maintenance [34, 35].

**Table 5:** Heterogeneity analysis results of the CESD score and the ADL score

	No chronic disease	Have chronic diseases	Low- education	High- education	Urban living	Rural living
Panel A. CESD score						
*Treat*_ic_x Post_t_	-1.0975^a^	-0.2221	-0.2850	-0.4440*	-0.2778	-0.4782
	(-1.7700)	(-1.0058)	(-0.8737)	(-1.8541)	(-1.0702)	(-1.5184)
Control variables	Yes	Yes	Yes	Yes	Yes	Yes
Adjusted R^2^	0.4753	0.5114	0.4762	0.5344	0.5278	0.4957
Sample Size	4396	20,646	13,197	12,083	9610	15,677
Panel B. ADL score						
* Treat*_ic_ x Post_t_	0.0221	-0.2288^c^	-0.2613^b^	-0.1691^b^	-0.0850	-0.3200^c^
	(0.1941)	(-3.3427)	(-2.5749)	(-2.5389)	(-1.0973)	(-3.4738)
Control variables	Yes	Yes	Yes	Yes	Yes	Yes
Adjusted R^2^	0.5473	0.5240	0.4999	0.5532	0.5672	0.5122
Sample Size	4447	21,034	13,533	12,188	9758	15,970

Note: Standard error for clustering to the individual level in parentheses; ^a^, ^b^, and ^c^ represent statistically significant at the 10%, 5%, and 1% level, respectively

This mechanism is also in line with the health production function logic: when preventive interventions become more accessible, the depreciation of health capital slows down, leading to improvements in both depressive symptoms and physical functioning. Moreover, enhanced community care intensity increases opportunities for older adults to access health support and social interactions, which facilitates psychological adjustment and functional maintenance [36]. The mechanisms are consistent with empirical findings, which show that the improvements in preventive behaviors significantly reduced depression and functional decline among older adults [37].

In conclusion, the empirical results provide clear support for the causal pathway“Pilot reforms lead to improved service accessibility, which in turn results in better health outcomes”, thereby strengthening both the theoretical interpretation and the external validity of this study.

### Heterogeneity analysis

The pilot reform of home and community elderly care services has a different impact on the health level of the elderly with different chronic disease conditions, education Level and urban and rural areas. This paper would analyze the heterogeneity of the impact of the pilot reform of home and community elderly care service on the health level of the elderly considering whether the elderly have chronic diseases, whether the elderly have low education or high education, and whether the elderly live in rural or urban areas. Results are shown in Table 6.

**Table 6:** Heterogeneity analysis results of the CESD score and the ADL score

	No chronicdisease	Havechronicdiseases	Low-education	High-education	Urbanliving	Ruralliving
Panel A. CESD score						
	-1.0975^a^	-0.2221	-0.2850	-0.4440^a^	-0.2778	-0.4782
	(-1.7700)	(-1.0058)	(-0.8737)	(-1.8541)	(-1.0702)	(-1.5184)
Control variables	Yes	Yes	Yes	Yes	Yes	Yes
Adjusted R^2^	0.4753	0.5114	0.4762	0.5344	0.5278	0.4957
Sample Size	4396	20646	13197	12083	9610	15677
Panel B. ADL score						
	0.0221	-0.2288^c^	-0.2613^b^	-0.1691^b^	-0.0850	-0.3200^c^
	(0.1941)	(-3.3427)	(-2.5749)	(-2.5389)	(-1.0973)	(-3.4738)
Control variables	Yes	Yes	Yes	Yes	Yes	Yes
Adjusted R^2^	0.5473	0.5240	0.4999	0.5532	0.5672	0.5122
Sample Size	4447	21034	13533	12188	9758	15970

Note: Standard error for clustering to the individual level in parentheses; ^a^, ^b^, and ^c^ represent statistically significant at the 10%, 5%, and 1% level, respectively

#### Disease Burden–Channel matching

Older adults with chronic conditions typically exhibit lower levels of functional health and have a more urgent demand for medical and rehabilitation services [38]. A series of reform measures—such as home visits, family doctor sign-ups, chronic disease follow-ups, and free health check-ups—provide effective support precisely tailored to these needs, thereby yielding significant improvements in functional health [39]. However, depressive symptoms in this group are often deeply influenced by factors such as pain, symptom unpredictability, and long-term disease burden. Consequently, short-term informational support or routine follow-up care may not lead to a substantial reduction in their CESD scores (a measure of depressive symptoms). In contrast, for older adults without chronic diseases, these reform measures primarily help alleviate health-related anxiety, confirm their risk status, and promote social integration, resulting in a marked improvement in their psychological state, as reflected by a notable decrease in CESD scores. Nevertheless, since the baseline functional health of this group is already near optimal, there is relatively limited room for further enhancement of their functional health.

#### Health literacy

The lower-education group showed a higher average ADL score of 1.39, while the higher-education group demonstrated a significantly better functional health status with a lower average ADL score of 0.78. Similarly, for depressive symptoms, the lower-education group had a higher average CESD score of 9.88, whereas the higher-education group exhibited a markedly lower average CESD score of 7.48, indicating substantially milder depressive symptoms.

The heterogeneity results by education level show that the policy had a stronger effect on functional health (ADL) among the low-education group (-0.2613, *p* < 0.05), while the improvement was smaller among the high-education group (-0.1691, *p* < 0.05). This suggests that older adults with lower education, who generally had poorer functional health and lower health literacy [40], benefited more from the increased availability of home- and community-based elderly care services. In contrast, the higher-education group already had a relatively better functional status, leaving less room for further improvement.

In terms of mental health, however, the policy effect on depressive symptoms (CESD) was larger and significant among the high-education group (-0.4440), while the effect was weaker and not statistically significant among the low-education group. It has been found that the improvement in mental health (CESD) resulting from the HCBS reform was more pronounced among older adults with higher education, whereas the mental health improvement among those with lower educational attainment was relatively limited. This phenomenon can be understood from three perspectives: cultural factors, health literacy, and structural differences. First, older adults with higher education generally place greater emphasis on mental health issues, are more inclined to proactively seek help, and participate in community activities, thereby making more effective use of the health education, information consulting, and social opportunities provided by HCBS [41]. In contrast, those with lower education may underutilize mental health resources due to cultural inertia or mental health stigma [42]. Second, disparities in health literacy also serve as an important mechanism. Older adults with higher education can more easily comprehend policy information and translate it into behavioral changes, such as actively engaging in community activities, accepting psychological counseling, and improving coping strategies, which directly enhances mental health outcomes [41, 42]. Third, structural disparities in resources further amplify this effect. Highly educated older adults often benefit from richer social networks and more opportunities for social participation, whereas those with lower education, especially in rural areas, face limitations in benefiting from the policy due to transportation barriers, limited community activities, and weaker social networks [43].

Therefore, although older adults with lower educational attainment might theoretically have more room for improvement in mental health, the disparities in cultural factors, health literacy, and structural barriers ultimately lead to their mental health improvements remaining limited and falling short of those observed in their highly educated counterparts. This underscores the necessity for policy designs to incorporate targeted psychological support and social participation interventions specifically for older adults with low education, in order to enhance mental health outcomes and achieve health equity.

#### Accessibility and baseline shortages

Descriptive statistics reveal pronounced urban-rural health inequalities, indicating that older adults in rural areas experience disadvantages in both physical and mental health. Specifically, rural older adults exhibit a significantly higher average CESD score (9.373 vs. 6.779) than city adults, reflecting more severe depressive symptoms and poorer mental health. Similarly, their average ADL score is higher (1.202 vs. 0.792), indicating worse functional health.

These disparities are not isolated but reflect broader systemic gaps in socioeconomic status and resource accessibility between urban and rural areas, which contribute to health inequities [44].

Heterogeneity analysis further demonstrates that the same policy intervention, such as the home- and community-based elderly care reform studied here, has significantly different effects on urban and rural older adults. In terms of functional health (ADL), the policy shows notable urban-rural heterogeneity: for urban older adults, the intervention did not significantly improve ADL (coefficient: -0.0850, insignificant), potentially due to their already better functional health (0.792). In contrast, the policy had a significantly positive effect on rural older adults (coefficient: -0.3200, significant at the 1% level), suggesting that the measure of policy effectively addressed long-standing issues in elderly care accessibility and met more urgent health needs in rural areas, resulting in more substantial improvements.

## Discussion

According to CHARLS 2011–2020 data and a multi-period DID design, this paper finds that the (HCBC) significantly improves both the mental and functional health of the elderly. The CESD scores decline by 0.396, ADL scores decline by 0.244, while the probability of using preventive care increases by 0.0252 (*p* < 0.01). In magnitude, these correspond to a reduction of approximately 4.53% relative to the sample mean for CESD, and a 20.1% reduction relative to the sample mean for ADL—both of which carry clear practical significance. These results confirm Hypothesis 1, the HCBS significantly improves elderly health outcomes, evidenced by reduced depressive symptoms (measured by the CESD scale) and diminished functional limitations in daily living (assessed by the ADL index).

In the mechanism, participation rates in home visits and family doctor contracts are significantly higher in the treatment group than in the control group (differences of 4.8 and 1.6% points, respectively). Moreover, the probability of “daily preventive care” rises by 0.0252, providing direct evidence for the channel “the policy enhances service accessibility, thereby improving health.” The findings are consistent with international literature showing that improved accessibility to elderly care services produces both functional and psychological benefits for older adults, which confirms Hypothesis 2 [34–37].

The heterogeneity analysis, among older adults without chronic conditions, the decline in depression is more pronounced (CESD: −1.0975*), while among those with chronic conditions, functional improvement is stronger (ADL: -0.2288***). In addition, CESD improvement is stronger among highly educated older adults (-0.4440*), whereas ADL improvement is greater among those with low education (-0.1691**). Finally, ADL improvements are concentrated in rural areas (-0.3200***). These results confirm Hypothesis 3, the HCBS improve mental health among chronic disease-free and highly-educated individuals, while enhancing functional health among chronic conditions, lower education, and rural elders. In fact, the health effects of the HCBS reform demonstrate a significant equity-oriented characteristic. The marginal gains in functional health from the policy intervention were greater among previously underserved groups with lower health and lower education status. This “equity catch-up effect” not only narrows the health disparities related to urban-rural divisions and educational attainment but also reflects a synergistic gain between efficiency and equity achieved by the policy. As noted in research on health equity, such as by [45], when public policies can improve the overall health level while simultaneously reducing structural inequalities, they generate social benefits that are far more profound than merely raising the average health status. The practical experience of the HCBS reform indicates that within the social aged care system, promoting institutional arrangements that unify accessibility, equity, and sustainability is a crucial pathway for achieving health equity and “common prosperity-type aging.

From the results, the HCBS is undoubtedly successful, and it has significantly improved the health levels of the elderly. From a macro perspective, the HCBS has achieved remarkable effectiveness and has charted a path for building an elderly care service system suited to China’s national conditions. The central government has adopted a scientific strategy of “pilots first, then scale-up.” Since 2016, China’s Ministries of Civil Affairs and Finance have selected 203 regions across the country in five batches to conduct HCBS reform pilots. To avoid “one-size-fits-all,” the central government has encouraged local governments to explore differentiated approaches based on their own ageing characteristics, economic foundations, and cultural traditions. As a result, numerous innovative models have emerged in areas such as community elderly care facilities, home-adaptation for older persons, smart elderly care platforms, and government-purchased services. More importantly, the central government has established an efficient mechanism for selecting, evaluating, and promoting outstanding pilot cases. After each batch of pilots completes, exemplary cases are chosen as national models, which greatly reduces the cost of system innovation, forming a virtuous cycle of “pilot exploration—experience summary—model cultivation—demonstration leadership,” thereby integrating effective central guidance with proactive local innovation.

Furthermore, the investment of 5 billion yuan in central fiscal funds has played a critical leveraging role. It has mobilised over 18 billion yuan from local governments and at least 13 billion yuan in social investment, for a total investment exceeding 36 billion yuan—realizing the original intention of small central input matched by large local and social capital input. This has laid a diversified and sustainable foundation for further funding. Building on the HCBS, many local governments have institutionalized the funding for the elderly service system construction into their annual fiscal budgets, and public welfare lottery funds have also been allocated proportionally, which has formed a stable mechanism for public investment. At the same time, the exploration and establishment of a long-term care insurance system has provided a dependable payment source for care services for disabled elders, and various governments have implemented fiscal and tax incentives, fee exemptions, or reductions for elderly service institutions, significantly reducing operating costs for social providers.

In addition, the success of the reform ultimately lies in empowering elderly individuals with precise, warm, and systematic services. Pilot regions across China have carried out detailed work around the goal “every elderly person should have access to basic elderly services.” Special and disadvantaged elderly people have been screened and identified so that the approach shifts from “people seeking services” to “services reaching people,” ensuring fairness and accessibility. Pilot regions have also experimented with establishing a standardized “basic community and home elderly care service catalogue,” defining service items and standards, thereby improving the normativity and transparency of service quality. Emergency response service networks and routine visit care for the disadvantaged elderly have also been constructed. According to incomplete statistics, more than 150 million person-times of elderly people have directly benefited from this reform; high satisfaction among recipients not only demonstrates expanded service coverage, but confirms that the content of services is precise and effective, earning genuine recognition from the people.

In summary, the HCBS has achieved decisive results in multiple dimensions: top-level design, financial leverage, service innovation, and the sense of benefit among the people.

Drawing on the HCBS experience and the current development landscape, the HCBS in the future should deepen toward greater precision, coordination, intelligence, and sustainability.

The core of the HCBS should lie in precisely identifying demand and optimizing service supply, which requires establishing a routine elderly capability assessment mechanism at the community level, dynamically tracking elderly people’s true needs, and forming a needs inventory. Against that baseline, elderly care service supply should shift from merely “having services or not” to “how well those services perform.” Emphasis should be placed on developing specialised care services aimed at older adults with chronic disease, disability, or dementia, and enriching diversified elderly care service offerings. An important direction is to promote ‘integrated operation of institution + community + home’, whereby the professional care resources of institutions radiate outward to serve the surrounding community elderly in their homes, achieving resource sharing.

Furthermore, technology will serve as a crucial support for enhancing the efficiency of the elderly care service system. In the future, the application of smart elderly care platforms should be comprehensively promoted to integrate various resources for elderly services, achieving full-process digital management from ‘demand profiling—intelligent service dispatch—service tracking—outcome evaluation.’ Simultaneously, it is essential to actively promote the use of smart wearable devices, emergency call systems, and remote monitoring equipment suitable for home environments to strengthen the safety monitoring of elderly individuals, particularly those living alone or at an advanced age. Utilizing big data analytics can also enable more accurate prediction of demand, optimize resource allocation, and realize the “digital-intelligent” upgrade of smart elderly care.

Besides, in the future, the HCBS should pay heightened attention to rural elderly care that is a glaring weak point. The main tasks are to accelerate the construction of a three-tier rural elderly care service network covering county, township, and village levels, and to extend high-quality urban elderly care resources outward into rural areas. The HCBS can explore mutual support models suitable for rural settings, encouraging younger, healthier elders to provide services to older, disabled elders. Simultaneously, the HCBS must be integrated with the Rural Revitalization Strategy. This involves revitalizing underutilized rural assets to establish village-level mutual support elderly care stations, thereby gradually narrowing the gap in the accessibility and quality of elderly care services between urban and rural areas.

Finally, a skilled workforce serves as the cornerstone for the development of the elderly care industry. In the future, it is essential to establish a comprehensive talent cultivation system integrating “academic education, vocational training, and continuing education,” regularly organize vocational skills competitions, and implement incentive mechanisms such as entry subsidies, post allowances, and professional career progression pathways. These measures aim to enhance the career attractiveness and social recognition of elderly care nurses.

Although this study employs panel data and a quasi-experimental design to systematically evaluate the effects of the HCBS and yields a series of positive findings, several limitations remain.

Measurement frequency and indicator limitations are major constraints. The study is mainly based on nationwide longitudinal datasets CHARLS, which tend to be conducted every 2–3 years, capturing medium to longer-term changes rather than short-term effects or instantaneous responses to reforms. Consequently, early impacts or transitional adjustments of the HCBS may have escaped detection. Moreover, although indicators such as ADL/IADL and depression scales are important measures of functional and psychological health, they may not capture all complexity, including cognitive decline, dementia, and sensory impairments, and are subject to self-reporting biases.

In addition, the issues of population representativeness and regional heterogeneity need to be solved. Although surveys like CHARLS cover a wide range of provinces and urban-rural areas, the sample sizes for certain specific groups, including older adults in extremely remote rural areas, empty-nesters/disabled older adults, and those with low income and very low education levels, are relatively small or the measurements are comparatively weak. These groups are often the most likely to be affected by inadequate policies and a lack of services. If the policy responsiveness of these groups differs from that of the main sample, the average effects estimated in this study might mask the true unmet needs and the room for improvement among these vulnerable populations. Furthermore, there are significant differences among local governments in terms of fiscal capacity, infrastructure, medical and aged care resources, and the degree of social organization participation. This regional heterogeneity may affect the outcomes of policy implementation. Although the models in this paper attempt to control for fixed effects or heterogeneity, it is difficult to fully isolate the influence of all locality-specific factors, such as health investments beyond local policies, local economic growth, etc., on the health outcomes.

Finally, service quality and implementation heterogeneity are not fully measured. Policy reform is not only about increasing service quantity or access, but critically depends on service quality. Yet the available data often record only ‘whether a service is provided’ or ‘participation/number of times’ rather than the quality, intensity, or fidelity of the service. Nor do they systematically track outcomes.

## Conclusion

In conclusion, the HCBC reform significantly improves both functional and psychological health among older adults by enhancing the accessibility of preventive and continuous services. The effect is supported by multiple robustness and identification checks, and exhibits interpretable heterogeneity across chronic disease status, education, and urban–rural divisions. Future governance should aim to move from universal coverage to population-specific customization and quality enhancement, thereby achieving healthy aging with higher cost-effectiveness.

At the policy level, the next stage of reform should focus on strengthening continuous follow-up, prescription and medication management, home environment and assistive device modifications, rehabilitation training, and in-home support for older adults with chronic conditions, low education, and in rural areas. Besides, the government should be embedding structured psychological and social participation programs for healthier and more educated groups. In addition, it should be improved in the closed-loop system of diagnosis, follow-up, referral, rehabilitation, and evaluation, with performance incentives guided by health outcomes and service continuity, and leveraging digital platforms for service tracking and assessment. Finally, the government would prioritize resource allocation toward functionally vulnerable and underserved areas to narrow gaps across regions and populations while amplifying overall health benefits.

## Supplementary Information

Supplementary Material 1

## Data Availability

I utilized the CHARLS database, which can be accessed and downloaded at the following URL: https://charls.charlsdata.com or https://charls.pku.edu.cn/en/Alternatively , you can search on Google, and the first result will be the website for the CHARLS database.In addition, you can obtain the list of home and community-based elderly care service reform pilot areas from the following official websites:1、https://www.shantou.gov.cn/stsmzj/gkmlpt/content/1/1387/post_1387752.html#3377 、https://news.cctv.com/2017/11/21/ARTIfGEjQN001DeVr92As45O171121.shtm、 https://www.mca.gov.cn/gdnps/pc/content.jsp?id=116521 、https://www.mca.gov.cn/n152/n165/c37127/content.html、https://www.mca.gov.cn/n152/n165/c38968/content.html.
