# Supplementary material for: The impact of the pilot reform of home and community-based elderly care services on the health of the elderly
Source: BMC Public Health. 2026 Jan 19;26:633. doi: 10.1186/s12889-026-26244-4 (PMC12911166; doi:10.1186/s12889-026-26244-4)
Supplement: Supplementary file 1 — Supplementary Material 1 [file 12889_2026_26244_MOESM1_ESM.pdf]

Appendix

Sensitivity analysis of parallel trends

The preceding analysis compared pre-treatment differences between groups to test the parallel trends assumption. However, recent studies indicate that pre-treatment trend tests are insufficient as empirical evidence for the parallel trends hypothesis[40]. Traditional parallel trend tests fail to effectively identify common pre-treatment trends between treated and control groups, potentially leading to biased estimation and inference[41]. In order to address this, this paper adopt the counterfactual approach for parallel trends proposed by Rambachan and Roth[42], which focuses on conducting inference and sensitivity analysis for post-treatment effect confidence intervals. Specifically, the parallel trends sensitivity analysis involves two steps: Firstly, constructing the maximum deviation degree (Mbar) from the parallel trends.Secondly, constructing confidence intervals for the post-treatment estimates corresponding to this deviation. If the confidence interval for the post-treatment point estimate excludes zero under the maximum Mbar scenario, the treatment effect is robust to deviations from parallel trends. Following Biasi and Sarsons[43], we set Mbar = 1 × standard error to test the sensitivity of the treatment effect to parallel trends violations after the pilot policy implementation.

Figure 3 shows that when only the "average relative magnitude of non-parallel trends" (Mbar) is relaxed, the robust confidence intervals for CESD and ADL remain entirely below zero within the feasible range, and the conclusions consistently remain significantly negative. Specifically, in panels (a) and (c), as Mbar gradually increases (up to 0.20 for CESD and 0.09 for ADL), the upper bound of the blue 95% robust interval never touches zero. This indicates that even if the treatment and control groups are allowed to deviate in their post-treatment average trends by as much as 20% of the maximum pre-trend deviation detected, the estimated policy effect remains statistically distinguishable from zero. Intuitively, the DID results are not valid merely because of the "strict parallel trends" assumption; rather, they remain robust even when allowing for considerable deviations (greater for CESD, somewhat smaller for ADL).

When relaxing the "smoothness restriction" (R), the robustness differs between the two health outcomes. Panel (b) shows that the robust interval for CESD gradually shifts upward as R increases, with its upper bound approaching zero at around R $\approx$ 0.18 – 0.20. In other words, only under an extreme setting where trend deviations are both relatively large and less smooth, the zero effect falls within the identified set. By contrast, panel (d) shows that the robust interval for ADL remains clearly below zero even when R is close to 0.10, indicating that the improvement in ADL is less sensitive to the smoothness relaxation and thus more robust. Taken together, the evidence suggests that under relaxations of relative deviation magnitude, both outcomes remain robust. Under more aggressive relaxations of smoothness, the significance of CESD becomes more fragile at the margin, but it still requires substantial relaxation to overturn the conclusions.

The implications are threefold. First, in conjunction with the baseline regressions in Table 2, the interval inference from HonestDiD supports the conclusion that the policy significantly reduces depressive symptoms and functional disability, and extends this significance from "point identification (strict parallel trends)" to "set identification (allowing measurable deviations)." Second, the ADL results are more robust under both

types of relaxation, suggesting that improvements in functional capacity are less associated with potential non-parallel trends and therefore provide stronger external validity and credibility. Third, for CESD, if reviewers are concerned about scenarios where post-treatment trends deviate substantially and lack smoothness, this study provides threshold evidence: only when R is relaxed to about 0.18 – 0.20 does the zero effect enter the identified set. This offers a clear counterfactual benchmark of "how much assumption relaxation would be required to overturn the conclusions.

Figure 3 Robustness Check: Applying Rambachan and Rotth(2023) Estimator

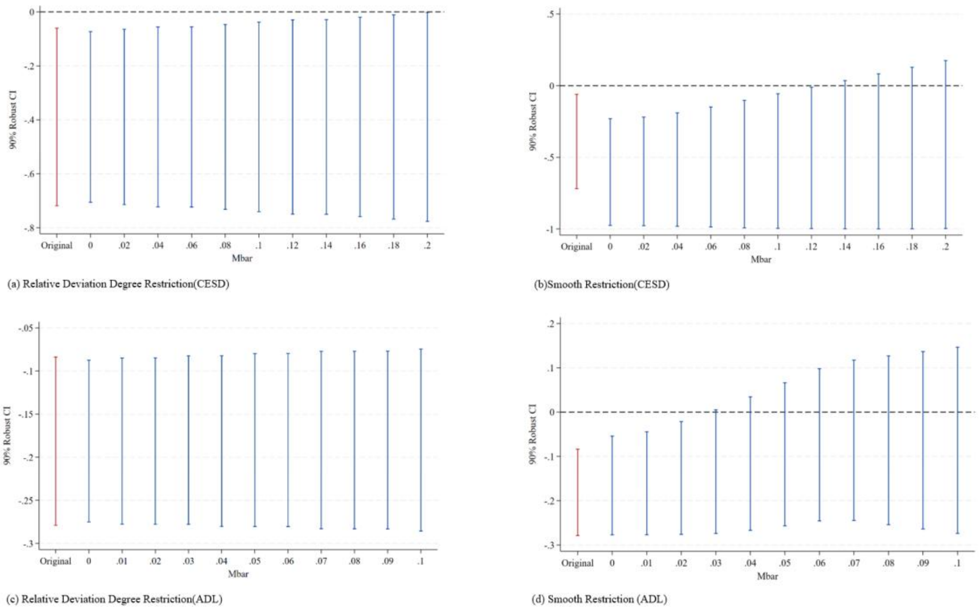

(a) Relative Deviation Degree Restriction(CESD)
(b)Smooth Restriction(CESD)
(c) Relative Deviation Degree Restriction(ADL)
(d) Smooth Restriction (ADL)

Bacon Analysis

In staggered DiD models under the two-way fixed effects (TWFE) framework, the core estimated coefficient represents a weighted average of canonical difference-in-differences estimators rather than the true treatment effect. Particularly when treatment effects exhibit heterogeneity across groups and time, staggered DiD estimators within the TWFE framework may suffer from substantial bias[44]. The bias in traditional two-way fixed effects (TWFE) estimators may arise from negative weights and heterogeneous treatment effects[45]. In order to rigorously examine the issue in the model, this study employs the Goodman-Bacon decomposition.

The TWFE (Two-Way Fixed Effects) point estimate to account for staggered adoption is decomposed into several 2 × 2 DID (Difference-in-Differences) comparisons. According to the Table 3 and Figure 4, for both CESD (depressive symptoms) and ADL (activities of daily living), the overall estimates are almost entirely driven by the "Treated vs. Never-Treated" comparison:The weights for this comparison are 0.960 for both outcomes. The corresponding ATTs (Average Treatment Effects) are −0.257 (CESD) and −0.223 (ADL) . Thus, the contribution of the "Treated vs. Never-Treated" unit to the overall estimate is approximately −0.247 (CESD) and −0.214 (ADL) . The weights for other comparisons are minimal.The weighted sum of the four sub-comparisons is −0.254 (CESD) and −0.217 (ADL) , consistent with the TWFE point estimates. This implies two key insights. Firstly, over 96% of the information in our

sample comes from the "clean" comparison of Treated vs. Never-Treated units. Cross-cohort comparisons between "Earlier-treated vs. Later-treated" (a potential source of bias) contribute negligibly. Secondly, the "Treated vs. Already-Treated" comparison shows a positive effect (+0.081) for CESD. This suggests that when "Already-Treated" units are mistakenly used as controls, the TWFE estimator may underestimate the true negative (improvement) effect of the treatment.

TABLE 3 Bacon Analysis

	CESD:Diff-in-diff estimate:-0.254		ADL: Diff-in-diff estimate: -0.217	
2×2-DID control type	Weights	Average treatment	Weights	Average treatment
Earlier T vs. Later C	0.007	-0.936	0.007	-0.364
Later T vs. Earlier C	0.003	-0.831	0.004	-0.010
T vs. Never treated	0.960	-0.257	0.960	-0.223
T vs. Already treated	0.030	0.081	0.029	-0.017

Note: T = Treatment; C = Comparison

Figure 4: Bacon Analysis(CESD and ADL)

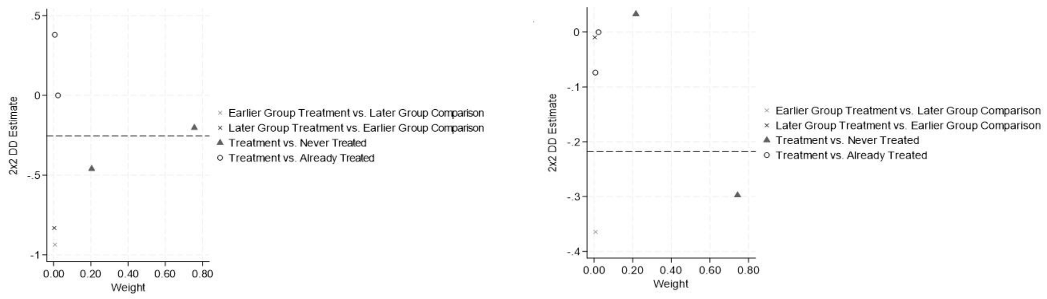

(a)Bacon Analysis(CESD) (b) Bacon Analysis(ADL)

Heterogeneity treatment effect

Based on the heterogeneity-robust DID estimators and methodologies, this paper employs the dynamic effect tests of the event study approach[46]and the stacked event study estimation[47]. Both sets of results corroborate the Bacon decomposition findings.

Across both CESD and ADL outcomes, the pre-treatment coefficients (t = −4 ⋯ −1) are close to zero with confidence intervals spanning zero, showing no evidence of systematic pre-trends. This strengthens the credibility of the parallel trends assumption.

As can be seen from the figure 5 and figure 6, In both estimators, the treatment effects turn negative immediately upon implementation (t = 0). For CESD, the effect deepens to approximately −1.3 by t = +1, while ADL declines to around −0.25, suggesting an immediate and progressively stronger improvement in psychological well-being and functional health.

The stacked event study estimator replicates the same downward trajectory as the heterogeneity-robust DID estimators, further confirming that the observed improvements are not artifacts of TWFE’s potential bias from heterogeneous adoption timing. This robustness indicates that the dynamic patterns of treatment effects are genuine and not sensitive to model specification.

Therefore, the health benefits of the HCBS are primarily captured by comparisons with never-treated units, minimizing bias from staggered adoption. Besides, the policy yields both immediate and increasing health gains over time. Importantly, the sharper decline in CESD relative to ADL suggests that psychological improvements emerge faster and more intensively, while functional health gains, though smaller in magnitude, remain highly robust.

Figure 5: Robustness Check: SA Estimator

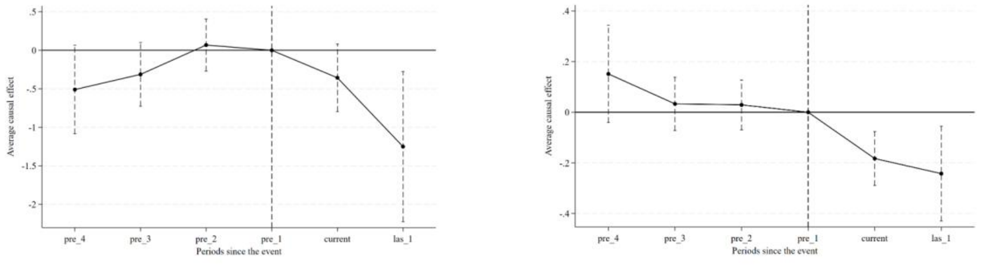

(a) SA Estimator(CESD)

(b) SA Estimator(ADL)

Figure 6:Robustness Check:Stackedev Estimator

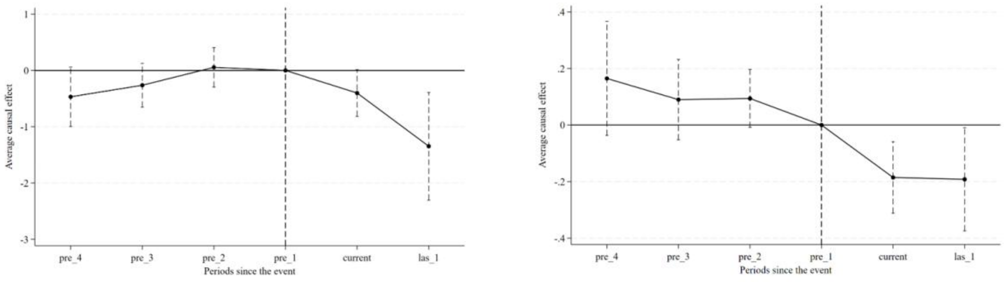

(a)Stackedev Estimator(CESD)

(b) Stackedev Estimator (ADL)

Placebo test

Considering the development level and policy promotion process of different cities, the characteristics of each city may potentially affect the empirical effect of this paper. In order to further verify the robustness and exclude the interference of other policies and random factors, a placebo test was conducted in this paper. The study examines whether the model estimates are affected by random factors, by simulating counterfactual scenarios through random assignment of the implementation timing of the home- and community-based elderly care service reform pilot policy. Specifically, if the estimates under random assignment are statistically insignificant, it indicates that the baseline results reflect genuine policy effects rather than random factors. This paper replicates the baseline regression 500 times to generate 500 parameter estimates. Figures 7 display the distribution of estimated coefficients for CESD and ADL scores across these 500 iterations. The results show that the mean coefficients for CESD and ADL scores are close to zero, the probability of estimated coefficients exceeding the true coefficients is below 1%, and the coefficient distributions follow a normal pattern. These findings confirm that the estimated results between treatment and control groups show no significant differences when the policy timing is randomized, demonstrating that the baseline regression results genuinely reflect the impact of the pilot policy on elderly health outcomes, further confirming the reliability of our estimates.

Figure 7: Placebo Test

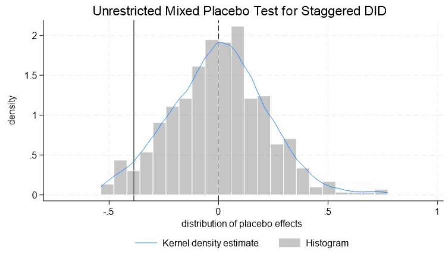

(a) Placebo Test(CESD)

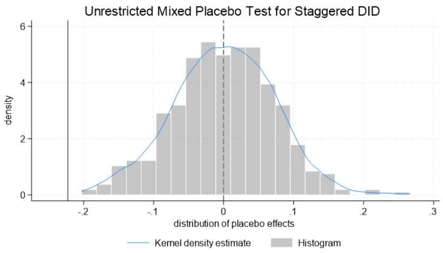

(b) Placebo Test(ADL)
